# The role of education attainment on 24-hour movement behavior in emerging adults: evidence from a population-based study

**DOI:** 10.3389/fpubh.2024.1197150

**Published:** 2024-01-16

**Authors:** Yanwei You, Leiyu Mo, Jing Tong, Xiangyu Chen, Yujun You

**Affiliations:** ^1^Division of Sports Science and Physical Education, Tsinghua University, Beijing, China; ^2^School of Social Sciences, Tsinghua University, Beijing, China; ^3^School of Law and Humanities, China University of Mining and Technology, Beijing, China; ^4^School of Educational Science, Harbin Normal University, Harbin, China; ^5^School of Educational Sciences, Yangzhou University, Yangzhou, China

**Keywords:** education level, sleep duration, work activity, exercise activity, sedentary behavior, emerging adults

## Abstract

**Purpose:**

The purpose of this study was to explore the relationship between education level and health behavior including sleep, work activity, exercise activity, and sedentary behavior among emerging adults.

**Methods:**

This study utilized data from the National Health and Nutrition Examination Survey (NHANES) collected between 2007 and 2018. The study sample included 4,484 emerging adults aged 18–25 years and the weighted participants were 30,057,813. Weighted multivariable regression analysis was performed to investigate the association between education level and the aforementioned health behavior, adjusting for age, gender, race/ethnicity, marital status, poverty-income ratio, BMI, smoking, and alcohol drinking status.

**Results:**

This study revealed that higher education level was associated with shorter sleep duration [Fully adjusted model, β (95% CI): −0.588 (−0.929, −0.246), *p* < 0.001]. Additionally, those with higher education levels were more likely to allocate time in sedentary behavior [β (95% CI): 90.162 (41.087, 139.238), *p* < 0.001]. Moreover, higher education level was related to less work activity [β (95% CI): −806.991 (−1,500.280, −113.703), *p* = 0.023] and more exercise activity time [β (95% CI): 118.196 (−21.992, 258.385), *p* = 0.097]. Subgroup analysis further verified this trend and detected that males with higher education level tended to participate in less work activity [β (95% CI): −1,139.972 (−2,136.707, −143.237), *p* = 0.026] while females with higher education level tended to engage in more exercise activity [Fully adjusted model, β (95% CI): 141.709 (45.468, 237.950), *p* = 0.004].

**Conclusion:**

This study highlighted the importance of education level as a significant factor in promoting healthy behavior among emerging adults. The findings underscored the need for the Ministry of Education to prioritize educating this demographic about the significance of maintaining adequate sleep patterns and reducing sedentary habits. Encouraging them to allocate more time for work and physical activities can significantly contribute to their overall wellbeing and success, ultimately fostering a healthier next generation.

## 1 Introduction

Education is a critical component of an individual's development, and it plays a significant role in shaping their future (1, 2). According to the Global Education Monitoring Report released by the United Nations Education Scientific and Cultural Organization (UNESCO), the global completion rate of primary school is 85%, that of junior high school is 73%, and that of senior high school is 49% (3). Despite the overall trend toward increased education among youth groups in recent years, there are still significant disparities in access to education, particularly between developed and developing countries, and between urban and rural areas (4–6). In addition, gender inequalities in education have also attracted attention and there is still a significant gender gap in education, particularly in developing countries (7, 8).

Within the prevailing paradigms of health behavior, the intricate nexus between education level and health reveals a causal relationship, as schooling may exert a transformative influence by augmenting the motivation and efficiency of health behavior. Running parallel to this notion, a supposition emerges, aligned with the observed positive correlation between schooling and health. This supposition heralds the concept of allocative efficiency (9). In accordance with this conjecture, erudite individuals exhibit a predilection for judiciously allocating health inputs, resulting in enhanced health, compared to their less educated counterparts. The proliferation of health-related knowledge induces a transformative metamorphosis within health behaviors, discernible in the patterns of consumption of health inputs, characterized by different products, encompassing domains such as medical care and physical activity, thereby exerting a discernible impact on health outcomes.

The relationship between schooling and health outcomes engenders a notable impact, whereby individuals with higher levels of education tend to exhibit superior health compared to their less educated counterparts (10). This association underscores the salience of educational attainment as a determinant of health disparities. Achieving high level education has an influence on the whole life span. A longitudinal national study found that in relatively low-educated individuals (< 12 years vs. >12 years of completed schooling), physical activity declined more steeply with aging process (11). Emerging adulthood is a critical period for promoting regular physical activity patterns, while it still remains uncertain whether education level at this stage influences such health behavior.

Emerging adult is a definition proposed by Jeffrey Jensen Arnett in American Psychologist (12). It refers to individuals between the ages of 18 and 25 who are transitioning from adolescence to adulthood (12, 13). This period is characterized by significant changes in various aspects of life, such as education, work, relationships, and personal identity, and is typically marked by a sense of exploration and experimentation. This period is a unique life stage characterized by significant transitions, such as leaving home, starting higher education or entering the workforce, and forming new social connections. These transitions can influence individuals' lifestyle choices and behaviors, including physical activity levels. Emerging adulthood is recognized as a distinct developmental stage, as individuals in this age group are no longer considered adolescents but have not yet fully transitioned into adult roles and responsibilities (14, 15). In a short time since the theory was used, a remarkable range of disciplines (including psychology, sociology, education, philosophy, epidemiology, and health sciences) have been interested in this issue (13). However, the education level in this targeted population and its role in health behaviors like sleep, physical activity, and sedentary behavior remain largely unknown.

According to 24-hour movement theory, indicators of young individuals' health are related to the composition of movement behavior through the day (sleep, work, exercise, and sedentary behavior) (16–18). A nationwide study has identified a short-sleep trend in recent decades (19). A study utilizing data from the United States National Longitudinal Study of Adolescent Health also found an age-related trend, with decreases across the adolescent period from 8.5 h per night to 7.3 h (20). Except for sleep, physical activity habits also significantly affect the health status of the young population. It has been shown that regular physical activity can improve mental health (21), enhance cognitive function (22), and promote good sleep (23), all of which can improve overall health status in emerging adults. Meanwhile, one study on the basis of Mendelian randomization detected that higher education may lead to more leisure-time physical activity (24). Nevertheless, the role of education in 24-hour movement behavior requires further investigation.

Collectively, limited and inconsistent evidence existed relating education level to 24-hour movement behavior, and it mostly concerned a small and specific group of people. With a nationwide sample from the National Health and Nutrition Examination Survey (NHANES) 2007–2018, we sought to: (1) identify the 24-hour movement behavior trend among emerging adults; (2) explore the relationship between education level and such health behavior in this population; and (3) use subgroup analysis to investigate the influence of gender on these associations.

## 2 Methods

### 2.1 Design and participants

Designed to assess Americans' health and nutritional status, the NHANES program examines the data of both adults and children. It is conducted by the Centers for Disease Control and Prevention's National Center for Health Statistics. NHANES has been conducted since 1999 to provide information on ~10,000 non-institutionalized household Americans over a 2-year period through a multistage probability sampling design. For the rationality of choosing this database, NHANES provides unique advantages in terms of its large sample size, standardized measures, and the ability to control for potential confounding factors. Moreover, the dataset's extensive coverage of demographic information, health behaviors, and health outcomes allows us to explore the associations between educational attainment and health behaviors comprehensively.

**Figure 1:**
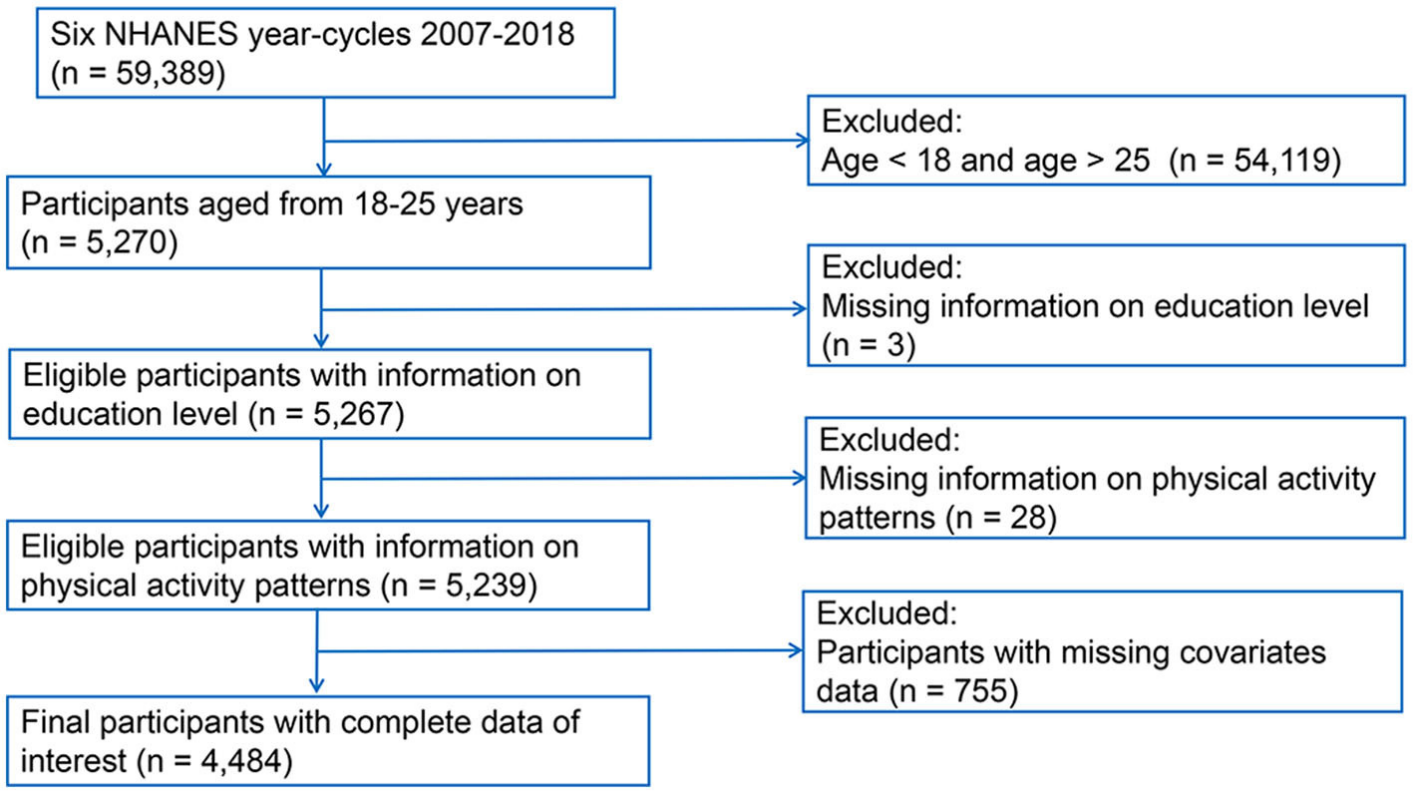
Detailed screening process for study participants.

After excluding participants < 18 and >25 years of age, a total of 5,270 participants were initially included in the analysis from six cycles of the “continuous NHANES” (2007/2008, 2009/2010, 2011/2012, 2013/2014, 2015/2016, 2017/2018). After that, participants without education information and 24-hour movement data were excluded, resulting in a calculated analytical cohort of 5,239 survey respondents. Finally, respondents lacking data records for covariates were eliminated from the analytical investigation, culminating in 4,484 participants for final analysis. The detailed screening process for study participants is provided in Figure 1.

### 2.2 Exposure and outcome measurements

The exposure variable in this study was the education level. To measure the education level of participants in the NHANES, a range of educational indicators and outcomes were assessed using a variety of validated and reliable instruments. Specifically, NHANES participants were asked to provide information about their educational history during a structured household interview. Referring to the previous literature (25, 26), education information was then recorded and categorized according to standard classifications of educational attainment, such as below high school, high school (some high school, or high school graduate), college or above (including the post-graduate education).

The outcome variables were 24-hour movement behaviors. Classic 24-hour movement behavior using accelerometer data is typically divided into physical activity, sedentary behavior, and sleep (27, 28). However, considering that our data are from questionnaires, we used self-reported work activity and exercise activity to represent the physical activity status. Referring to previous research (29–31), work activity refers to physical activity or exertion related to one's occupation or job. It encompasses movements and efforts made during work-related tasks, including standing, walking, lifting, or any physical exertion associated with one's job responsibilities. Moreover, exercise activity usually pertains to planned, structured, and repetitive recreational activities performed during leisure time for the primary purpose of improving or maintaining physical fitness and health. It includes activities such as jogging, cycling, swimming, sports participation, and gym workouts.

To capture both the frequency and intensity of work and exercise activity in NHANES, the information of physical activity was self-reported by the Physical Activity Questionnaire using a 7-day recall method. Details about the physical activity questionnaire can be found at the NHANES website. Participants provided self-reports regarding the frequency and duration of each distinct form of physical activity, for both moderate and vigorous intensities, in a typical week. Subsequently, the sum of the minutes of physical activity for each intensity was computed to derive the total minutes of work and recreational activity, respectively, as the final outcome measure. Sleep duration was determined by asking participants how much sleep they typically get per day. “How much sleep do you get (hours) each day?” Additionally, the duration of sedentary behavior per day was evaluated utilizing a query that inquired “What is the typical duration of time you spend engaged in prolonged sitting on an average day (minutes)?”

### 2.3 Covariate assessment

In conducting an analytical inquiry utilizing the NHANES database, it was of the utmost importance to account for potential covariates that may impact the results. Referring to several previous reports (32–34), the study controlled for confounding factors such as age, gender, race/ethnicity, marital status, family poverty income ratio, body mass index (BMI), smoking and alcohol drinking status. This rigorous approach to covariate assessment underscored the importance of accounting for potential confounders in NHANES-based investigations and highlighted the need for precise and thorough methodology in research utilizing this valuable resource. Based on the responses provided within the administered questionnaire, the smoking status of respondents was characterized as never smoking, former smoking or current smoking. Concurrently, the alcohol usage profile of the participants was stratified into three distinct classifications: non-drinking, moderate alcohol drinking, and high alcohol drinking. In accordance with established literature (35), individuals classified as moderate drinkers were characterized by a weekly alcohol intake of 14 or fewer drinks for male, or 7 or fewer drinks for female. Additionally, the threshold for heavy/excessive drinking were defined as more than 14 drinks/week for male or more than 7 drinks/week for female or 5 or more drinks/day in a single day.

### 2.4 Statistical analysis

In accordance with the established NHANES guidelines, the collation of all relevant data was performed, whereupon it was subjected to analysis utilizing the survey weighting methodology. The NHANES website offered guidance on employing complex multistage sampling methods, weighted analyses, and adjusting for selection probabilities through strategies like weighting, clustering, and stratification. These statistical methods, integrated into the analysis using the “survey” package in R, aimed to provide nationally representative estimates by accommodating survey weights and the intricacies of the survey's design. More detailed guidelines can be found at: https://wwwn.cdc.gov/nchs/nhanes/analyticguidelines.aspx.

The mobile examination center interview weights were employed for the purpose of addressing non-response, non-coverage, and the existence of dissimilar probabilities of selection, with the objective of amalgamating survey data collected over a 12-year period spanning from 2007 to 2018. The merged weights (WT) were calculated as WT = (1/6) ^*^ WTMEC2YR_07/08_ + (1/6) ^*^ WTMEC2YR_09/10_ + (1/6) ^*^ WTMEC2YR_11/12_ + (1/2) ^*^ WTMEC2YR_13/14_ + (1/6) ^*^ WTMEC2YR_15/16_ + (1/6) ^*^ WTMEC2YR_17/18_ +, where WTMEC2YRs were variables from NHANES 2007–2018.

In the present investigation, we employed both unadjusted and adjusted models (36, 37). The crude model remained unadjusted for any covariates. Model 1, on the other hand, was adjusted for age, gender, and race. Model 2, the fully adjusted model, was further controlled for marital status, poverty status, body mass index, smoking status, and alcohol drinking status. A weighted linear regression model was employed to investigate the relationship between educational attainment and 24-hour movement behavior in this study. Furthermore, subgroup analyses were performed to explore the potential impact of gender on such relationships. All statistical analyses were conducted using the R Foundation's software (http://www.R-project.org), and a *p*-value of 0.05 or less was considered statistically significant.

## 3 Results

### 3.1 Population characteristics

**Figure 2:**
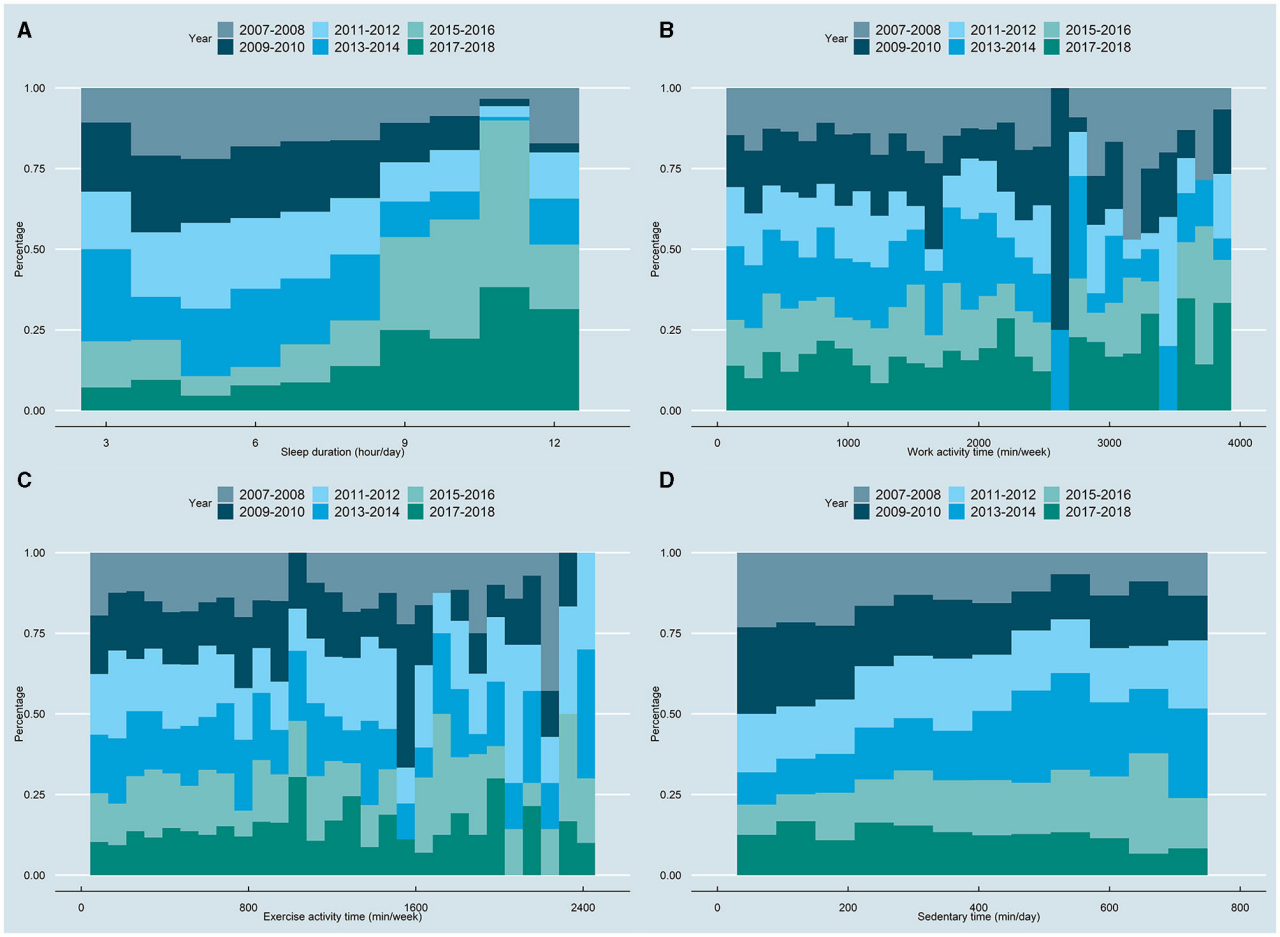
Histogram percentage distribution of 24-hour movement behavior in different years, **(A)** sleep duration; **(B)** work activity; **(C)** exercise activity; **(D)** sedentary behavior.

The present investigation encompassed a sample of 4,484 emerging adults aged 18–25, with a weighted population of 30,057,813, constituting the final analytical cohort. Table 1 conveys the sociodemographic characteristics of the enrolled individuals. The mean age of the study population was 21.17 years, with a male-female ratio of 0.98. The average duration of sleep among the subjects was determined to be 7.44 h per day [Q1 (≤ 6): 26.94%, Q2 (>6, ≤ 7): 23.60%, Q3 (>7, ≤ 8): 29.08%, Q4 (>8): 20.38%], which conformed to the recommended 7–8 h of sleep per day by the National Sleep Foundation (38). Moreover, the mean weekly work and exercise activities were observed to be 890.85 min [Q1 (≤ 1): 51.61%, Q2 (>1, ≤ 840): 23.73%, Q3 (>840): 24.67%] and 402.11 min [Q1 (≤ 1): 36.78%, Q2 (>1, ≤ 150): 13.31%, Q3 (>150, ≤ 540): 25.74%, Q4 (>540): 24.17%], respectively, while the average duration of sedentary time was 362.65 min per day [Q1 (≤ 240): 35.12%, Q2 (>240, ≤ 360): 25.83%, Q3 (>360, ≤ 480): 19.09%, Q4 (>480): 19.96%]. In regard to the temporal trend of the distribution of the four health behaviors, although no significant alterations were discerned in work and exercise activities, the analysis revealed that participants in the recent 2015–2016 and 2017–2018 cycles tended to exhibit longer sleep durations and shorter sedentary times (Figure 2).

**Table 1:** Weighted characteristics of emerging adults in the NHANES (2007–2018) by education attainment.

**Variable**	**All participants**	**Below high school**	**High school**	**College or above**	***P*-value**
**Age**					< 0.001
< 22	46.56	27.39	64.32	27.55	
22–24	27.27	33.22	18.52	36.79	
≥24	26.17	39.39	17.16	35.66	
**Sex**					< 0.001
Male	50.55	59.09	54.84	45.36	
Female	49.45	40.91	45.16	54.64	
**Race/ethnicity**					< 0.001
Non-hispanic White	58.02	31.85	55	62.56	
Non-hispanic Black	13.68	6.73	15.12	12.39	
Mexican American	12.4	41.27	15.31	7.85	
Other Race/ethnicity	15.9	20.16	14.57	17.21	
**Marital status**					< 0.001
Never married	73.45	45.21	76.98	70.77	
Married/living with partner	24.87	51.11	21.6	27.35	
Widowed/divorced	1.68	3.68	1.42	1.89	
**Poverty-income ratio**					< 0.001
< 1	28.81	56.63	32.12	23.86	
1–3	40.08	41.48	39.97	40.14	
≥3	31.11	1.89	27.91	35.99	
Body mass index (kg/m^2^)					0.224
< 25	48.91	42.85	49.05	49.03	
25–30	24.69	32.83	23.44	25.72	
≥30	26.4	24.31	27.51	25.25	
**Smoking status**					< 0.001
Never smoking	72.57	57.03	70.96	75.07	
Former smoking	8.5	14.94	7.33	9.54	
Current smoking	18.93	28.03	21.72	15.4	
**Alcohol drinking status**					< 0.001
Non-drinking	30.87	43.29	41.06	18.91	
Moderate alcohol drinking	35.21	24.72	28.2	43.53	
High alcohol drinking	33.92	31.99	30.74	37.56	
Sleep duration (hour)	7.44 ± 0.03	7.88 ± 0.15	7.52 ± 0.04	7.32 ± 0.04	< 0.001
Work activity time (min/week)	890.85 ± 39.29	1,625.24 ± 357.86	1,016.36 ± 50.17	717.42 ± 48.71	< 0.001
Exercise activity time (min/week)	402.11 ± 16.55	251.44 ± 67.75	398.92 ± 16.92	412.44 ± 23.3	0.084
Sedentary time (min/day)	362.65 ± 4.82	274.05 ± 22.56	338.87 ± 5.39	393.25 ± 6.82	< 0.001

For continuous variables: survey-weighted mean ± SE, P-value was calculated by survey-weighted linear regression. For categorical variables: survey-weighted percentage (%), P-value was calculated by survey-weighted Chi-square test.

By using weighted linear regression models, the relationship between education attainment and 24-hour movement behavior was examined. Taking the below high school group as a reference, Table 2 reveals that high school and college or above education level was associated with a decrease of 0.417 and 0.588 h of sleep per day [High school, β (95% CI): −0.417 (−0.749, −0.084), *p* = 0.015; College or above, β (95% CI): −0.588 (−0.929, −0.246), *p* < 0.001] in the fully adjusted model. Education level was also associated with less work activity time [High school, β (95% CI): −445.369 (−1,161.684, 270.946), *p* = 0.219; College or above, β (95% CI): −806.991 (−1,500.280, −113.703), *p* = 0.023] and exercise activity time [High school, β (95% CI): 76.267 (−58.831, 211.364), *p* = 0.264; College or above, β (95% CI): 118.196 (−21.992, 258.385), *p* = 0.097]. This indicated that compared with the below high school group, high school and college or above education level were associated with a decrease of 445.369 and 806.992 min per week of work activity. Similarly, compared with the reference group, high school and college or above education level groups were associated with an increase of 76.267 and 118.196 min per week of exercise activity. Moreover, it was identified that higher education level was related to more sedentary time [High school, β (95% CI): 36.968 (−9.097, 83.033), *p* = 0.114; College or above, β (95% CI): 90.162 (41.087, 139.238), *p* < 0.001].

**Table 2:** Weighted linear regression results of the relationship between education attainment and 24-hour movement behavior.

	**Crude model**		**Model 1**		**Model 2**	
	**β (95% CI)**	***P*-value**	**β (95% CI)**	***P*-value**	**β (95% CI)**	***P*-value**
**Sleep duration (hour/day)**						
Below high school	Reference		Reference		Reference	
High school	−0.352 (−0.673, −0.031)	0.032	−0.371 (−0.687, −0.054)	0.022	−0.417 (−0.749, −0.084)	0.015
College or above	−0.556 (−0.884, −0.228)	0.001	−0.517 (−0.844, −0.190)	0.002	−0.588 (−0.929, −0.246)	< 0.001
**Work activity time (min/week)**						
Below high school	Reference		Reference		Reference	
High school	−608.874 (−1,342.125, 124.378)	0.103	−492.05 (−1,202.166, 218.066)	0.172	−445.369 (−1,161.684, 270.946)	0.219
College or above	−907.821 (−1,615.748, −199.893)	0.013	−875.634 (−1,563.787, −187.481)	0.013	−806.991 (−1,500.280, −113.703)	0.023
**Exercise activity time (min/week)**						
Below high school	Reference		Reference		Reference	
High school	147.481 (5.329, 289.632)	0.042	86.348 (−44.986, 217.683)	0.195	76.267 (−58.831, 211.364)	0.264
College or above	161.002 (18.457, 303.547)	0.027	172.717 (36.190, 309.244)	0.014	118.196 (−21.992, 258.385)	0.097
**Sedentary time (min/day)**						
Below high school	Reference		Reference		Reference	
High school	64.816 (20.806, 108.826)	0.004	50.781 (5.912, 95.650)	0.027	36.968 (−9.097, 83.033)	0.114
College or above	119.197 (71.386, 167.009)	< 0.001	106.117 (57.128, 155.106)	< 0.001	90.162 (41.087, 139.238)	< 0.001

Crude model, no covariates were adjusted. Model 1, age, gender, and race/ethnicity were adjusted. Model 2, age, gender, race/ethnicity, marital status, poverty status, body mass index, smoking status, and alcohol drinking status were adjusted.

**Figure 3:**
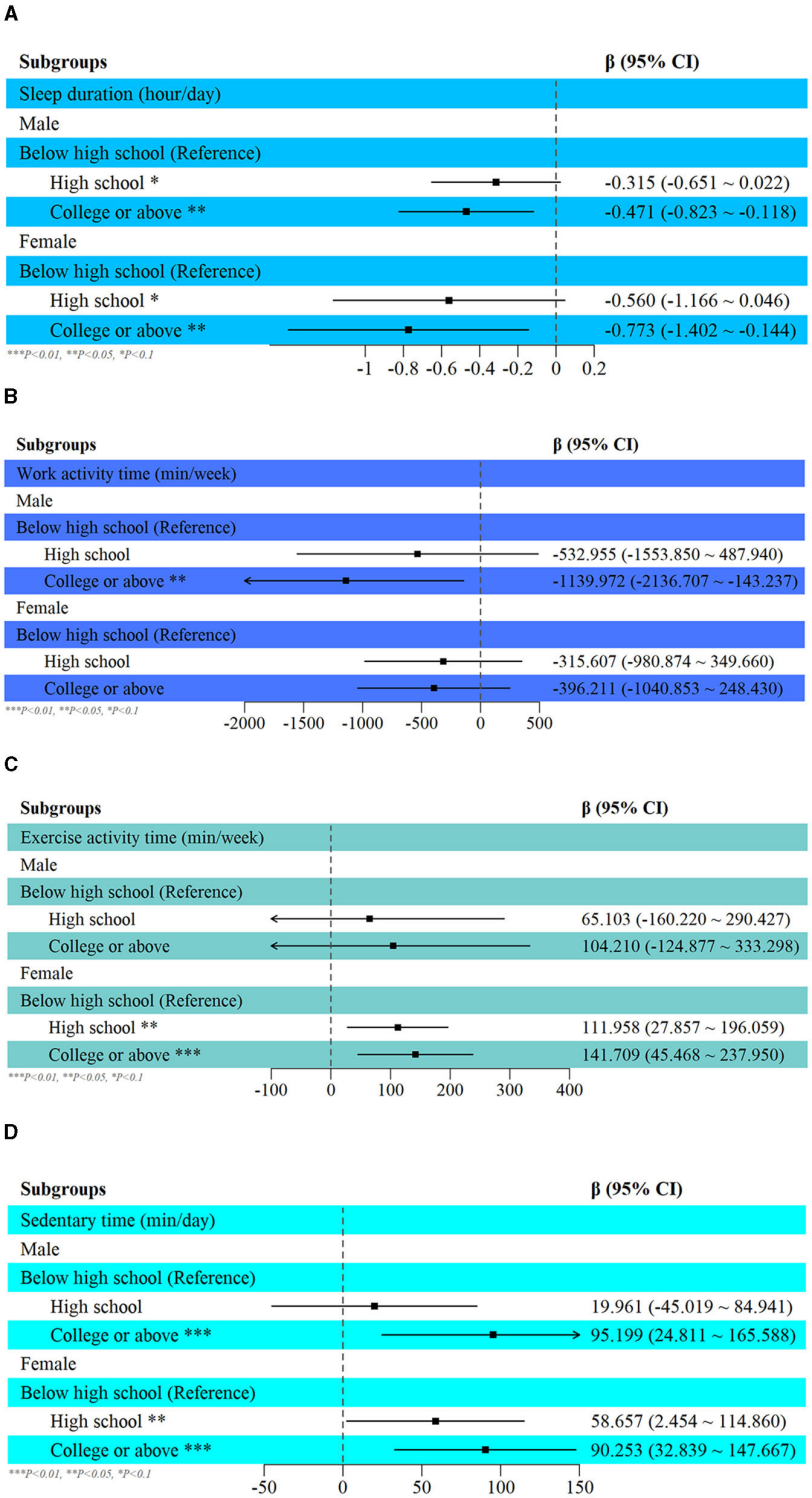
Subgroup analysis of the relationship between education attainment and 24-hour movement behavior in the male and female group, **(A)** sleep duration; **(B)** work activity; **(C)** exercise activity; **(D)** sedentary behavior.

In addition, subgroup analysis demonstrated that these associations were generally consistent across males and females (Figure 3). Results demonstrated a consistent negative association between education attainment reaching collage or above and sleep duration in both male and female groups [for males, β (95% CI): −0.471 (−0.823, −0.118), *p* = 0.010; for females, β (95% CI): −0.773 (−1.402, –0.144), *p* = 0.017]. Additionally, an upward trend was identified between higher education and sedentary behavior [For males, β (95% CI): 95.199 (24.811, 165.588), *p* = 0.009; For females, β (95% CI): 90.253 (32.839, 147.667), *p* = 0.002]. For work activity, however, a negative relationship was found in the male group [For males, β (95% CI): −1,139.972 (−2,136.707, −143.237), *p* = 0.026] while no significant correlation was found in the female group [For females, β (95% CI): −396.211 (−1,040.853, 248.430), *p* = 0.225]. When it comes to exercise activity, no significant association was found in the male group [For males, β (95% CI): 104.21 (−124.877, 333.298), *p* = 0.368] while a positive association persisted in the female group [For females, β (95% CI): 141.709 (45.468, 237.950), *p* = 0.004]. More details of subgroup analysis were described in Supplementary Table 1.

## 4 Discussion

Exploring the relationship between educational attainment and health behavior such as sleep, physical (work and exercise) activity, and sedentary behavior has important implications for both the education and health fields. The current study, based on a sample analysis of 4,484 emerging adults from NHANES 2007–2018, reported the association between education attainment and 24-hour movement behavior for the first time and verified the gender influence on this relationship.

There is a growing concern about the impact of education levels on emerging adults' sleep patterns. Our studies showed that as education levels increase, the amount of time spent sleeping decreases. Several studies conducted in different regions reported that higher education levels may be associated with more time spent on academic work and extracurricular activities, and associated with less time for sleep. Additionally, higher education levels may be associated with increased stress and anxiety, which can negatively impact sleep quality and duration (39–41). Moreover, cultural expectations surrounding academic success and achievement may lead adolescents with higher education levels to prioritize academic work and career development over adequate sleep (42, 43). To promote this group's health, it is important to prioritize sleep hygiene and promote healthy sleep habits among all adolescents, regardless of their education levels. This includes providing education on the importance of sleep, encouraging consistent sleep routines, and promoting the avoidance of technology and social media use before bedtime.

The relationship between education and physical labor is complex and multifaceted, especially among emerging adults. While higher levels of education are often associated with decreased physical labor, there are also benefits to engaging in physical labor, such as improved physical health and skill development (44). However, it should also be noted that excess physical labor may also associated with higher risks of musculoskeletal symptoms (45). Gender differences in 24-hour movement behavior have been a subject of considerable interest and research. This study identified a negative association between education level and work activity in the male group, while this trend was not significant in the female group. For the potential explanation of gender differences, girls are more likely to drop out of school, and less likely to attend school in the first place due to societal and cultural barriers (46, 47). One study conducted in Spain found that males have a predilection for adhering more closely to prescribed physical activity guidelines compared to their female counterparts. Conversely, females tend to exhibit a higher degree of adherence to recommendations related to screen time limitations (48). However, it's important to note that these differences are often observed as trends across populations and might not apply universally to every individual.

There is a growing concern about the negative effects of sedentary behavior on youth's health, particularly as it relates to education levels. It was observed that as education levels increase, so does the amount of time spent in sedentary behavior among emerging adults. This relationship is particularly pronounced among those achieving college degrees or above, who may spend extended periods of time sitting in class, studying, or working on computers (49). One possible explanation for this trend is that higher education levels often require more time spent on academic work, which may involve prolonged periods of sitting. Moreover, higher education may provide access to more technology and sedentary activities (50, 51), such as video games and social media.

To mitigate the negative effects of sedentary behavior, it is a necessity to promote physical activity and healthy lifestyles among emerging adults (52). This includes promoting active transportation to and from school (53), providing opportunities for physical activity during school hours (54), and encouraging participation in different types of exercise (55). In this study, an overall positive relationship was identified between education level and exercise activity, which demonstrated that emerging adults who complete higher levels of education are more likely to engage in physical exercise than those who are below high school education. One reason for this relationship is that higher levels of education provide adolescents with access to information about the benefits of physical activity and the skills necessary to engage in physical activity (56). Additionally, emerging adults who complete higher levels of education may also have more opportunities to participate in organized sports and physical activity programs, as well as access to facilities such as gyms and sports clubs.

It is imperative to note that this study has multiple strengths. To the extent of our knowledge, this was the first study that reported the relationship between education level and health behavior among emerging adults. Due to the multistage probability sampling design of NHANES, this study can offer a better generalizability of the US emerging adult population. As a result of the large sample size, we were able to perform further analyses on subgroups. There are, however, some limitations to be considered. Firstly, education attainment and 24-hour movement behavior cannot be determined causally due to this study's cross-sectional design. Second, physical activity patterns were assessed by the questionnaire, which may lead to subjective reporting bias. Additionally, due to the characteristic of the NHANES data, physical activity was measured as a “typical week” measure, while sleep and sedentary behaviors were assessed “per day.” This may cause differences in time frames for measuring physical activity, sleep, and sedentary behaviors. Third, although our study specifically focuses on the U.S. population, longitudinal follow-up studies conducted in different regions such as both the developing and developed countries will be needed to verify these findings. Last but not least, this study only focused on the influence of education level. Further studies should explore the relevance of disparities in access to education.

## 5 Conclusion

In conclusion, this study explored the association between education attainment and health behavior in US emerging adults using a nationwide population. The increased education level was associated with shorter sleep duration and more sedentary time. Moreover, participants with higher education tended to conduct less work activity and achieve more exercise activity. By promoting healthy physical activity habits among emerging adults, schools, parents, and healthcare providers can help to improve academic performance and reduce the risk of health problems associated with short sleep and sedentary behavior. By prioritizing adequate sleep, appropriate physical activity and less sedentary time, emerging adults can improve their overall health and wellbeing, and set themselves up for success both now and in the future.

## Data availability statement

The original contributions presented in the study are included in the article/Supplementary material, further inquiries can be directed to the corresponding author.

## Author contributions

YaY, LM, and JT: study conception and design, data collection, data analysis, and drafting of manuscript. YaY, LM, JT, XC, and YuY: interpretation of results. YaY, XC, and YuY: providing valuable insight regarding the approach and organization of the manuscript. YuY: supervision. All authors provided critical revisions of the draft and approved the submitted draft.
