# Supplementary material for: The role of education attainment on 24-hour movement behavior in emerging adults: evidence from a population-based study
Source: Front Public Health. 2024 Jan 16;12:1197150. doi: 10.3389/fpubh.2024.1197150 (PMC10824836; doi:10.3389/fpubh.2024.1197150)
Supplement: Supplementary file 1 [file Table_1.docx]

Table S1. Subgroup analysis of associations between education attainment and physical activity patterns by different genders.

|  | Male | | Female | |
| --- | --- | --- | --- | --- |
|  | β (95% CI) | *P-value* | β (95% CI) | *P-value* |
| **Sleep duration (hour/day)** |  |  |  |  |
| Below high school | Reference |  | Reference |  |
| High school | -0.315(-0.651, 0.022) | 0.066 | -0.560(-1.166, 0.046) | 0.069 |
| College or above | -0.471(-0.823,-0.118) | 0.010 | -0.773(-1.402,-0.144) | 0.017 |
| **Work activity time (min/week)** |  |  |  |  |
| Below high school | Reference |  | Reference |  |
| High school | -532.955(-1553.850, 487.940) | 0.302 | -315.607( -980.874, 349.660) | 0.348 |
| College or above | -1139.972(-2136.707,-143.237) | 0.026 | -396.211(-1040.853, 248.430) | 0.225 |
| **Exercise activity time (min/week)** |  |  |  |  |
| Below high school | Reference |  | Reference |  |
| High school | 65.103(-160.220, 290.427) | 0.567 | 111.958( 27.857,196.059) | 0.010 |
| College or above | 104.21(-124.877, 333.298) | 0.368 | 141.709( 45.468,237.950) | 0.004 |
| **Sedentary time (min/day)** |  |  |  |  |
| Below high school | Reference |  | Reference |  |
| High school | 19.961( -45.019, 84.941) | 0.543 | 58.657( 2.454,114.860) | 0.041 |
| College or above | 95.199( 24.811,165.588) | 0.009 | 90.253( 32.839,147.667) | 0.002 |

Notes: Fully adjusted model was used. Age, race/ethnicity, marital status, poverty status, body mass index, smoking status, alcohol drinking status were adjusted.
